# The Neuroprotective Mechanism of Low-Frequency rTMS on Nigral Dopaminergic Neurons of Parkinson's Disease Model Mice

**DOI:** 10.1155/2015/564095

**Published:** 2015-03-25

**Authors:** Qiaoyun Dong, Yanyong Wang, Ping Gu, Rusheng Shao, Li Zhao, Xiqi Liu, Zhanqiang Wang, Mingwei Wang

**Affiliations:** ^1^Fifth Department of Neurology, Cangzhou Central Hospital, No. 16 Xinhua Western Road, Cangzhou, Hebei 061000, China; ^2^Hebei Province Key Laboratory of Brain Aging and Cognitive Neuroscience, No. 16 Xinhua Western Road, Cangzhou, Hebei 061000, China; ^3^Department of Neurology, Cangzhou Central Hospital, No. 16 Xinhua Western Road, Cangzhou, Hebei 061000, China

## Abstract

*Background*. Parkinson's disease is a neurodegenerative disease in elder people, pathophysiologic basis of which is the severe deficiency of dopamine in the striatum. The purpose of the present study was to evaluate the neuroprotective effect of low-frequency rTMS on Parkinson's disease in model mice. *Methods*. The effects of low-frequency rTMS on the motor function, cortex excitability, neurochemistry, and neurohistopathology of MPTP-induced Parkinson's disease mice were investigated through behavioral detection, electrophysiologic technique, high performance liquid chromatography-electrochemical detection, immunohistochemical staining, and western blot. *Results*. Low-frequency rTMS could improve the motor coordination impairment of Parkinson's disease mice: the resting motor threshold significantly decreased in the Parkinson's disease mice; the degeneration of nigral dopaminergic neuron and the expression of tyrosine hydroxylase were significantly improved by low-frequency rTMS; moreover, the expressions of brain derived neurotrophic factor and glial cell line derived neurotrophic factor were also improved by low-frequency rTMS. *Conclusions*. Low-frequency rTMS had a neuroprotective effect on the nigral dopaminergic neuron which might be due to the improved expressions of brain derived neurotrophic factor and glial cell line-derived neurotrophic factor. The present study provided a theoretical basis for the application of low-frequency rTMS in the clinical treatment and recovery of Parkinson's disease.

## 1. Introduction

Parkinson's disease (PD) is a major neurodegenerative disease in elderly population, the symptom of which includes bradykinesia, resting tremor, muscular rigidity, and gait disturbance [1, 2]. The onset and development of PD is found to be closely related to the cell loss and eosinophilic intracytoplasmic aggregation in substantia nigra (SN) [3]. Recent studies have reported a significant decrease of the striatal dopamine (DA) in PD patients [4] and the conclusion was supported by that most degenerated cells in SN expressed proteins involved in the synthesis, degeneration, and transport of DA [5, 6]. The underlying mechanism of this neuron loss in PD patients remains unrevealed; however, hypothesis that the degeneration of nigral dopaminergic neuron (NDN) is linked to a lack of trophic support was proposed. Those trophic factors include brain derived neurotrophic factor (BDNF) and glial cell line derived neurotrophic factor (GDNF), which have been reported to be downregulated in the SN from PD patients. Therapeutic methods focusing on increasing the level of BDNF and GDNF in the SNare are being developed.

Despite the fact that the nigral DA cell loss has been taken as a specific characteristic of PD, the etiology of PD is still not well understood, which makes it difficult to develop perfect therapeutic strategy. Traditional therapies such as DA precursor and operation can only contribute to the control of the disorder but fail to prevent neuronal degeneration along with lots of side effects [7, 8]. Development of promising alternative therapeutic strategies is imperative.

In recent studies, it was suggested that repetitive transcranial magnetic stimulation (rTMS) might be a potential treatment for different neuropsychiatric diseases [9–13]. The technique is a novel noninvasive and painless treatment associated with few or mild side effects which can produce lasting changes in excitability as well as activity in cortical regions and functionally connected cortical or subcortical regions [9–13]. Several studies have showed that high-frequency (>1 Hz) and low-frequency (<1 Hz) rTMS both had a beneficial effect on motor functions in PD patients [11, 12]; moreover, low-frequency rTMS had a higher safety in clinical practice than high-frequency rTMS. Rationales of rTMS on PD involve the increasing release of DA and BDNF in certain brain areas [14]. However, the mechanism through which rTMS exerts therapeutic effect stays unclear.

In the present study, we investigated the effects of low-frequency rTMS on motor function, cortex excitability, neurochemistry, neurohistopathology of 1-methyl-4-phenyl-1,2,3,6-tetrahydropyridine- (MPTP-) induced PD mice by behavioral detection, electrophysiologic technique, high performance liquid chromatography-electrochemical detection (HPLC-ECD), immunohistochemical staining, and western blot. We hoped that our study would reveal the mechanism underlying the effect of low-frequency rTMS on PD and improve the practical application of low-frequency rTMS in treating PD patients in the future.

## 2. Materials and Methods

### 2.1. PD Model Establishment and Low-Frequency rTMS Treatment

Fifty-six eight-week-old male C57BL/6j mice weighing 240–260 g were randomly divided into four groups, 14 mice for each group: NS group (control group, healthy mice receiving normal saline group injection), PD group (acute PD model group, mice receiving MPTP injection to establish acute PD model), s-rTMS group (sham group, PD model mice exposed to the noise during the rTMS stimulation), and rTMS group (low-frequency rTMS treatment group, PD mice receiving low-frequency rTMS stimulation). For PD model establishment, the mice received four MPTP injections (15 mg/kg, s.c., dissolved in 0.9% saline) with two-hour intervals at the 1st day of the experiment to establish an acute PD model while the mice in NS group were injected with the same volume of saline instead of MPTP at the same time points. All animal experiments were conducted in the accordance with the Institutional Animal Ethics Committee and Animal Care Guidelines of Hebei Medical University that governed the use of experimental animals.

Twenty-four hours after the last injection, the mice in rTMS group received five trains of 1 Hz stimulation for 25 s at the intensity of 1 Tesla with a 10 mm diameter circular coil. The interval between each strain was 2 min. Centers of the coils were 15 mm higher than mice heads. During the stimulation, all the animals were awakened. The treatment was conducted one time per day for mice in rTMS group and lasted for 14 consecutive days at the same time point each day. Mice in s-rTMS group were exposed to the same noise during the rTMS simulation for 14 days with center of coils being more than 10 cm apart from the mice heads. No treatment was performed on mice in NS and PD groups.

### 2.2. Effect of Low-Frequency rTMS on the Behavior of PD Model Mice

The mice were assessed for the motor movement using an automated locomotor activity test and a rotarod test before agents injections and at 1st, 3rd, 7th, and 14th day after the last injection. For automated locomotor activity test, the observation was conducted using a ZH-YLS-ZA Automated Locomotor Activity Control Instrument (Huaibeizhenghua Biological Instrument, Co., LTD) according to the user introduction. Rotary test was conducted based on the method of Vijitruth et al. [15]: mice were placed in a 20 cm diameter rotating bar, and the number of the rotation before mice left the bar was recorded. Each mouse was tested for five times at each time point and the interval between each time was 5 min.

### 2.3. The Effect of Low-Frequency rTMS on the Cortex Excitability of PD Model Mice

Thirty-two eight-week-old male C57BL/6j mice weighing 240–260 g were randomly divided into four experimental groups as described above, 8 mice for each group. Treatments were performed as described above. Then mice were assessed for their resting motor threshold (RMT) according to previous studies [16, 17] before agents injection and at 1st, 3rd, 7th, and 14th day after the last injection: mice were anesthetized with 40 mg/kg chloralhydrate and stimulated using Magpro X100 Magnetic Stimulator (Dantec Dynamics A/S, Denmark) by placing coil at left brain of the mice. RMT values of the hind leg gastrocnemius were recorded by collecting the magnetic stimulation signal using Counterpoint Electromyography (Oxford Instruments, UK).

### 2.4. Effect of Low-Frequency rTMS on the Level of DA and Its Metabolites in the Striatum of PD Model Mice

Twenty-four male C57BL/6J mice weighing 240–260 g were randomly divided into four experimental groups as described above, 6 mice for each group. Treatments were performed on each group as described above. 24 h after the last stimulation of low-frequency rTMS, mice in each group were executed by breaking the necks, and the level of DA, homovanillic acid (HVA), and 3,4-dihydroxyphenylacetic acid (DOPAC) in mice striatum was detected using high performance liquid chromatography-electrochemical detection (HPLC-ECD) following standard procedures in Beijing Institute of Neurosciences, Capital Medical University.

### 2.5. Effect of Low-Frequency rTMS on the Function of NDN of PD Model Mice

Fifty-six male C57BL/6J mice weighing 240–260 g were randomly divided into four experimental groups as described above, 14 mice for each group. Treatments were performed on each group as described above. 24 h after the last stimulation of low-frequency rTMS, eight mice from each group were anesthetized with 40 mg/kg chloralhydrate, perfused with 30 mL normal saline and 100 mL 40 g/L paraformaldehyde solution, and brain samples containing SN were then quickly frozen and cut into sections for immunohistochemical staining of tyrosine hydroxylase (TH), BDNF, and GDNF proteins in the SN: samples from each group were fixed with 3% hydrogen peroxide and blocked with 10% normal goat serum for 60 min. They were then incubated separately with primary rabbit antibodies against TH (1 : 5000, Chemicon), BDNF (1 : 100 ABcam), and GDNF (1 : 15, ABcam) at 4°C overnight. Samples were rewarmed for 60 min at 37°C before incubating with goat anti-rabbit IgG (1 : 100, Neomarker) for another 60 min at 37°C. Then samples were incubated with horseradish peroxidase (HRP, 1 : 100) for 60 min at 37°C and then with DAB-H_2_O_2_ for 5 min to develop color. Observation of the immunohistochemical staining was conducted using a microscope under 100x magnification. Corrected optical density (COD) value was calculated as the difference between OD values of immunoreactive cells and those of pyramidal tracts cells.

Fresh tissue was dissected from the SN of midbrain of the left six mice in each group after cervical dislocation. Total protein was extracted for TH, BDNF, and GDNF detection by western blot following standard procedures: the extracts were boiled with loading buffer for five minutes and then subject to sodium dodecyl sulfate polyacrylamide gel electrophoresis (SDS-PAGE) on 10% gels. Targeted proteins were transferred onto polyvinylidene difluoride sheets. The membranes were washed with TBST for three times, 20 min for each time. Then the membranes were incubated with primary antibody (rabbit antibody against TH (1 : 1000, Chemicon); rabbit antibody against BDNF (1 : 500, Chemicon); rabbit antibody against GDNF (1 : 25, ABcam); rabbit antibody against *β*-actin (1 : 300, ABcam)) overnight at room temperature. After additional three washes, secondary antibody (goat anti-rabbit IgG (1 : 1000, Neomarker)) was added and the membrane was incubated for four hours. After three final washes, the blots were developed using Beyo ECL Plus reagent and the results were detected in the Gel Imaging System. The content of the targeted protein was expressed as relative optical densities (RODs), which were calculated as the ratio of OD values of the targeted proteins to that of *β*-actin detected by Quantity One software (Bio-Rad Laboratories, Inc.).

### 2.6. Statistical Analysis

All the data were expressed in the form of mean ± SD. Multiple comparisons were conducted by *q* method and correlation was calculated using Pearson coefficiency. All the statistical analyses were conducted using SPSS version 16.0 (IBM, Armonk, NY, USA) with a significant level being 0.05.

## 3. Results

### 3.1. Low-Frequency rTMS Treatment Improved the Behavior of PD Model Mice

**Figure 1:**
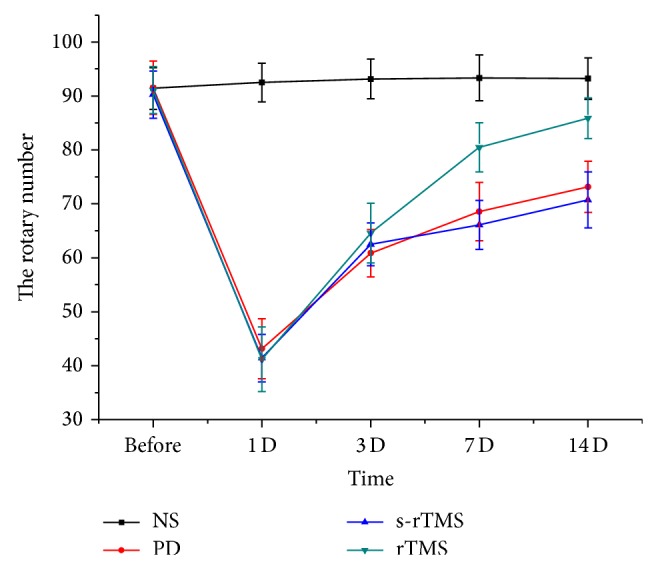
The comparison of rotarod testing in different groups at different time points.

**Figure 2:**
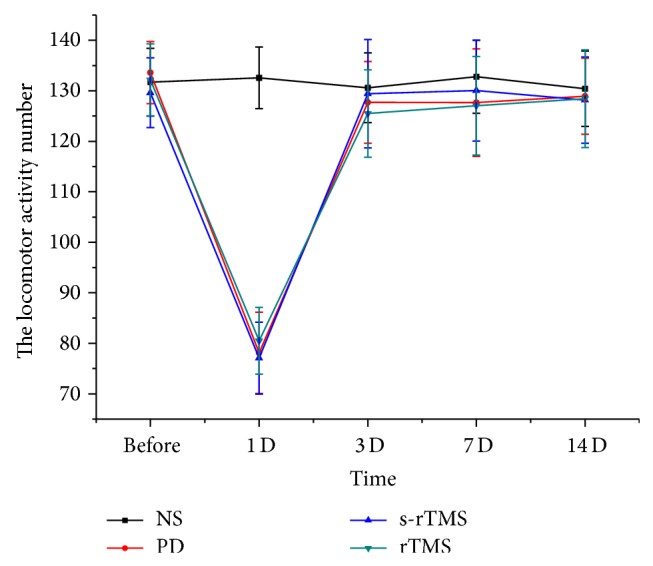
The comparison of the locomotor activity in different groups at different time points.

Shortly after the first injection of MPTP, model mice showed performance of piloerection, bradykinesia, stroub tail reaction, instability of gait, tremor, and the toes of the hind feet widely separated. Those performances remained in MPTP-treated mice for 5 to 6 h. Similar symptoms were observed after the subsequent injections, and the symptoms of bradykinesia, stiffness, and instability of gait strengthened with the injection of MPTP. However, all the mice showed recovery as regards external appearance 24 h after injection. No abnormality was observed in the control group. No dysfunction was observed in the stimulated animals during or after low-frequency rTMS. The results of automated locomotor activity test showed that treatment of low-frequency rTMS had no improvement on the locomotor activity of model mice (Figure 1; See Supplementary Table S1 in Supplementary Material available online at http://dx.doi.org/10.1155/2015/564095); however, significant effect of low-frequency rTMS on the improvement of rotary number was observed in rotary test (Figure 2; Table S2).

### 3.2. Low-Frequency rTMS Treatment Improved the Cortex Excitability of PD Model Mice

**Figure 3:**
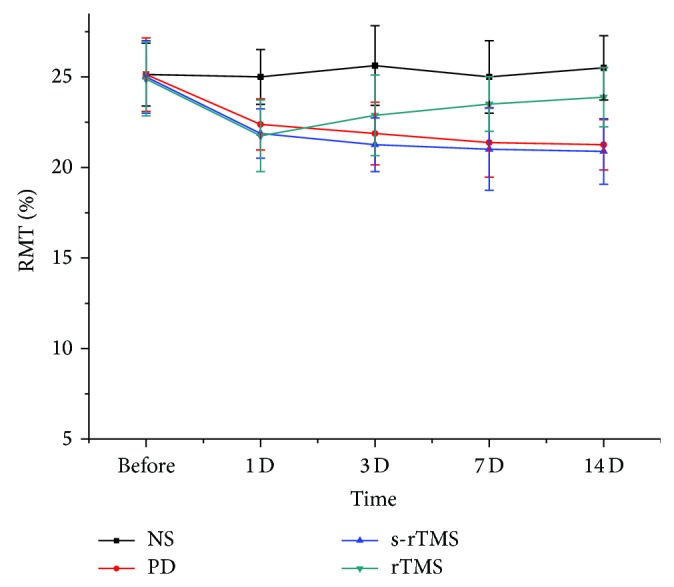
The comparison of RMT of mice in different groups at different time points.

Significant decreases of the RMT of mice were observed in MPTP-treated groups compared to that of the NS group after the first injection of MPTP (Figure 3; Table S3) (*P* < 0.05). The decrease persisted for PD and s-rTMS groups for the left injections but with a mild pattern. For rTMS group, increase of the RMT of mice was observed since the 3rd day after the last injection. The difference of RMT between rTMS group and PD/s-rTMS groups was significant at the 7th day and 14th day (Figure 3; Table S3) (*P* < 0.05).

### 3.3. Low-Frequency rTMS Increased the Level of DA and Related Metabolites in the Striatum of PD Model Mice

**Figure 4:**
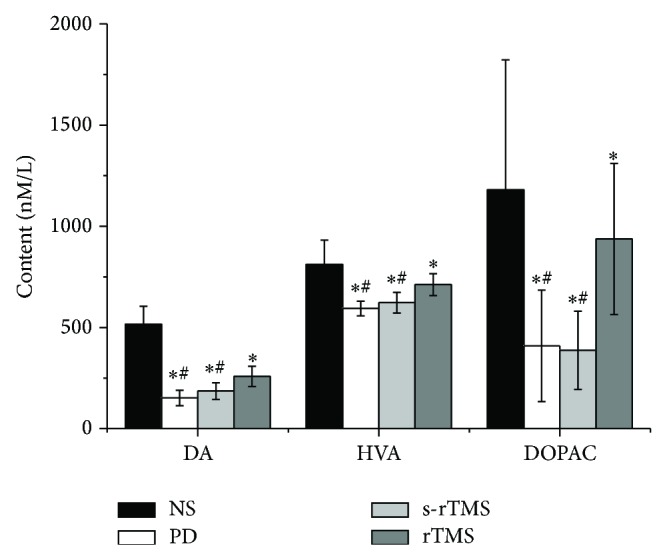
The effects of low-frequency rTMS on the content of DA and its metabolites in the striatum of PD model mice. ^*^Significantly different from NS group, *P* < 0.05; ^#^significantly different from rTMS group, *P* < 0.05.

A dramatic decline of DA level was observed in the striatum of PD mice (Figure 4; Table S4) (*P* < 0.05). DOPAC and HVA level also decreased compared to NS group (Figure 4; Table S4) (*P* < 0.05). After low-frequency rTMS treatment, the DA, DOPAC, and HVA levels in rTMS group were significantly increased compared with PD and s-TMS groups (Figure 4; Table S4) (*P* < 0.05). No significant difference was found between s-rTMS and PD group (Figure 4; Table S4).

### 3.4. The Protective Effect of Low-Frequency rTMS on the NDN of PD Model Mice

**Figure 5:**
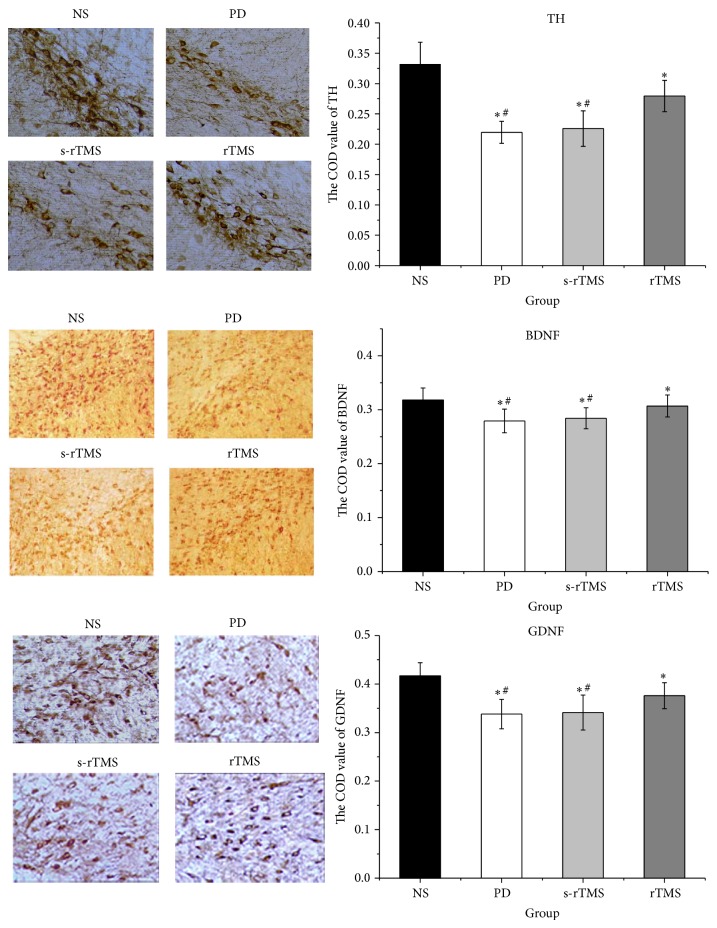
The effect of low-frequency rTMS on the expression of TH, BDNF, and GDNF as illustrated by immunohistochemical staining. ^*^Significantly different from NS group, *P* < 0.05; ^#^significantly different from rTMS group, *P* < 0.05.

**Figure 6:**
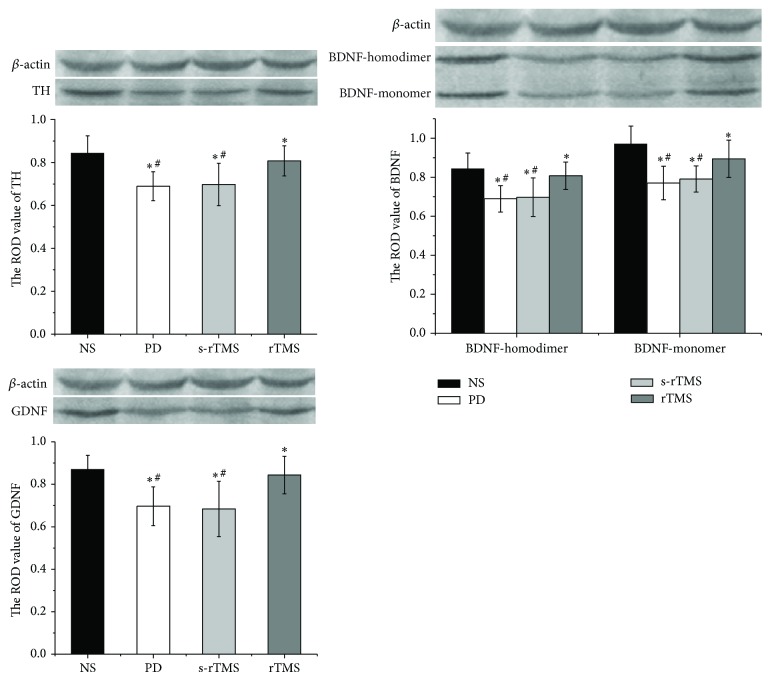
The effect of low-frequency rTMS on the expression of TH, BDNF, and GDNF as illustrated by western blot. ^*^Significantly different from NS group, *P* < 0.05; ^#^significantly different from rTMS group, *P* < 0.05.

The results of immunohistochemical staining were shown in Figure 5 and Table S5. Generally, the expressions of TH, BDNF (including two bands: monomer and homodimer), and GDNF all significantly declined in PD model mice. However, the symptoms were reversed after treatment of low-frequency rTMS. And the results showed that the changes of COD of TH were positively related to those of BDNF and GDNF (Table 1). Same patterns were also observed in the western blot of TH, BDNF, and GDNF, which confirmed the protective effect of low-frequency rTMS on the NDN of PD model mice (Figure 6; Tables 2 and S6).

## 4. Discussions

PD is thought to be a result of complex rearrangements in the neuronal circuits which is responsible for motor activity [18–20]. The study of Fox et al. [21] firstly described the application of rTMS in treating PD, which reported that subthreshold, high-frequency (5 Hz) rTMS over M1 induced a dramatic improvement of reaction and movements times, as well as the performance on the grooved pegboard test. These benefits were confirmed by other groups [22–24]. In the present, we focused on the therapeutic effect of low-frequency rTMS on PD for its higher safety in clinical practice compared to high-frequency rTMS.

Although, in the behavior test, locomotor activity improvement of model mice was not related to low-frequency rTMS treatment, significant improvement of rotary number was observed in the rTMS group (Figures 1 and 2). Such results should suggest that low-frequency rTMS acts on a subclinical level and is strongly effective in normalizing endophenotypic abnormalities of the MPTP induced PD models but has not yet the sufficient efficacy to fully restore even behavior impairment. In addition, the effect of low-frequency rTMS on rotary number increased with the time of treatment, showing the accumulative effect of therapy. Low-frequency rTMS could also act on the cortex excitability of PD model mice and increased of the RMT of model mice since the 3rd day after the last MPTP injection. The difference of RMT between rTMS group and PD/s-rTMS groups was significant at the 7th day and 14th day (Figure 3). Previous studies showed that high-frequency rTMS could increase the excitability of motor cortex [25], while rTMS lower than 1 Hz could significantly reduce the excitability of cortex with a longer effect [26]. Kinds of brain circuits in PD patients were blocked resulting from the inefficient synthesis of DA. By changing the local excitability and activity of neuron, low-frequency rTMS could influence the long distant cortex and subcortical area and reverse the abnormality in different brain circuits. In addition, the effect of low-frequency rTMS on RMT also had an accumulative effect (Figure 3), which did not show significant difference from those of PD and s-rTMS group until the 7th day of the treatment.

The injection of MPTP could significantly decline the synthesis of DA and its metabolites in the PD model mice (Figure 4). Most of DA distributed in SN striate system, and the main metabolites of DA were HVA and DOPAC. Those molecules all play important roles in motion control [27] and content of DA in basal ganglia declining to 30% of normal level would cause clinical symptoms of PD [28]. Compared with PD and s-rTMS groups, the syntheses of DA, HVA, and DOPAC were much higher in rTMS group (Figure 4).

Previous study has also indicated that effect of rTMS function is not only through the activation of cortex of PD but also by influencing basal ganglia through striatum [29]. This conclusion was consistent with our results, in which the levels of TH, BDNF, and GDNF were all strengthened in rTMS group (Figures 5 and 6). TH has been taken as the marker enzyme of neurons producing dopamine in brain [30]. The increase of TH would directly improve the synthesis of DA.

Recent studies have proposed that the degeneration of SN may be due to a lack of neurotrophic factor [31], and the influence of rTMS on the expression of BDNF and GDNF could lead to the protective effect of NDN. BDNF has a critical role in cell differentiation, neuronal survival, migration, dendritic arborization, synaptogenesis, and synaptic plasticity [32]. GDNF promotes the growth, regeneration, and survival of SN dopamine neurons. Both of the two neurotrophins have been reported to decease in PD [33–36]. Results of immunohistochemical staining and western blot showed that the content of BDNF and GDNF dramatically declined in model mice, but the expressions were all improved significantly after a consecutive treatment of low-frequency rTMS (Figures 5 and 6).

Although our study has provided some evidence for the effect of low-frequency rTMS on the protection of NDN, other reports have also revealed the same effect of high-frequency rTMS treatment. In a study of Lee et al. [37], it was reported that chronic 10 Hz rTMS enhanced the expression of BDNF, GDNF, and increased DA positive neurons in rat model of PD. Based on the results of both studies, it seemed that the protective effect of rTMS might not be due to the frequency. Given the higher safety of low-frequency rTMS, we recommended that application of low-frequency rTMS was the first option in clinical treatment of neurodegenerative disease such as PD.

## 5. Conclusions

In conclusion, our results showed that low-frequency rTMS had a considerable effect on the physiological characteristics of MPTP induced PD model mice and could be a potential therapy of PD. The mechanism might be due to the neuroprotective effect on NDN by the improved expressions of BDNF and GDNF. And we suggested that further studies on low-frequency rTMS in treating PD should be conducted. Systematical evaluation of the relevant methodological features would facilitate the establishment of more prominent and longer lasting effects of the therapy.

## Supplementary Material

Raw data of automated locomotor activity test, rotary test, RMT value , HPLC-ECD detection, immunohistochemical staining, and western blot.

## Figures and Tables

**Table 1:** Correlation analysis between COD values of TH and BDNF or GDNF as illustrated by immunohistochemical staining. in SN of mice.

	Groups	Correlation Coefficient	*P* value
TH and BDNF	NS	0.906	0.002
	PD	0.894	0.003
	s-rTMS	0.903	0.002
	rTMS	0.967	0.000

TH and GDNF	NS	0.959	0.000
	PD	0.898	0.002
	s-rTMS	0.730	0.040
	rTMS	0.919	0.001

**Table 2:** Correlation analysis between ROD values of TH and BDNF/GDNF as illustrated by western blot.

	Groups	Correlation Coefficient	*P* value
TH and BDNF-monomer	NS	0.887	0.018
	PD	0.872	0.024
	s-rTMS	0.858	0.029
	rTMS	0.820	0.046

TH and BDNF-homodimor	NS	0.828	0.042
	PD	0.898	0.015
	s-rTMS	0.842	0.036
	rTMS	0.839	0.037

TH and GDNF	NS	0.889	0.018
	PD	0.845	0.034
	s-rTMS	0.908	0.012
	rTMS	0.824	0.044

## Abbreviation

PD: Parkinson's disease
SN: Substantia nigra
DA: Dopamine
NDN: Nigral dopaminergic neuron
BDNF: Brain derived neurotrophic factor
GDNF: Glial cell line-derived neurotrophic factor
rTMS: Repetitive transcranial magnetic stimulation
COD: Corrected optical density
ROD: Relative optical density.
