# Supplementary material for: The Neuroprotective Mechanism of Low-Frequency rTMS on Nigral Dopaminergic Neurons of Parkinson's Disease Model Mice
Source: Parkinsons Dis. 2015 Mar 25;2015:564095. doi: 10.1155/2015/564095 (PMC4390107; doi:10.1155/2015/564095)
Supplement: Supplementary file 1 — Raw data of automated locomotor activity test, rotary test, RMT value , HPLC-ECD detection, immunohistochemical staining, and western blot. [file 564095.f1.pdf]

Table S1 Raw data of automated locomotor activity test.

Group	Day				
	Before	1D	3D	7D	14D
NS	131.7±6.7	132.6±6.1	130.6±6.9	132.8±7.2	130.4±7.5
PD	133.6±6.2	78.1±8.0*	127.7±8.1*	127.7±10.6	128.9±7.6
s-rTMS	129.6±6.9	77.1±7.1*	129.4±10.7*	130.0±10.0	128.2±8.5
rTMS	132.2±7.1	80.5±6.6*	125.5±8.6*	127.0±9.8	128.4±9.7

*, significantly different from NS group, $P < 0.01$

Table S2 Raw data of rotary number.

Group	Day				
	Before	1D	3D	7D	14D
NS	91.4±3.9	92.5±3.6	93.1±3.7	93.3±4.2	93.2±3.8
PD	91.5±5.0	43.1±5.5*	60.8±4.4*	68.6±5.4*#	73.1±4.7*#
s-rTMS	90.3±4.4	41.4±4.4*	62.5±4.0*	66.1±4.5*#	70.7±5.2*#
rTMS	91.1±4.3	41.2±6.0*	64.6±5.5*	80.5±4.6*	85.9±3.7*

*, significantly different from NS group, $P < 0.05$; #, significantly different from rTMS group, $P < 0.05$

Table S3 Raw data of RMT value

Group	Day				
	Before	1D	3D	7D	14D
NS	25.1±1.7	25.0±1.5	25.6±2.2	25.0±2.0	25.5±1.8
PD	25.1±2.0	22.4±1.4*	21.9±1.7*	21.4±1.9*#	21.3±1.4*#
s-rTMS	25.0±2.0	21.9±1.4*	21.3±1.5*	21.0±2.3*#	20.9±1.8*#
rTMS	24.9±2.0	21.8±2.0*	22.9±2.2*	23.5±1.5*	23.9±1.6*

*, significantly different from NS group, $P < 0.05$; #, significantly different from rTMS group, $P < 0.05$

Table S4 Raw data of HPLC-ECD detection

Group	DA (nM/L)	HVA (nM/L)	DOPAC (nM/L)
NS	515.4±90.0	809.9±121.9	1180.1±641.2
PD	151.3±38.5*#	593.3±36.45*#	409.0±275.6*#
s-rTMS	186.0±40.7*#	622.39±51.0*#	386.8±193.0*#
rTMS	258.2±50.4*	711.9±54.0*	936.8±373.6*

*, significantly different from NS group, $P < 0.05$; #, significantly different from rTMS group, $P < 0.05$

Table S5 Raw data of COD values in immunohistochemical staining.

Group	TH	BDNF	GDNF
NS	0.33±0.04	0.32±0.02	0.42±0.03
PD	0.22±0.02*#	0.28±0.02*#	0.34±0.03*#
s-rTMS	0.23±0.03*#	0.28±0.02*#	0.34±0.04*#
rTMS	0.28±0.03*	0.31±0.02*	0.38±0.03*

*, significantly different from NS group, $P < 0.05$; #, significantly different from rTMS group, $P < 0.05$

Table S6 Raw data of COD values in western blot.

Group	TH	BDNF-homodimer	BDNF-monomer	GDNF
NS	0.84±0.08	0.97±0.07	0.99±0.07	0.87±0.07
PD	0.69±0.07*#	0.82±0.04*#	0.82±0.04*#	0.70±0.10*#
s-rTMS	0.70±0.10*#	0.79±0.06*#	0.79±0.06*#	0.68±0.13*#
rTMS	0.81±0.07*	0.91±0.10*	0.91±0.10*	0.84±0.09*

*, significantly different from NS group, $P < 0.05$; #, significantly different from rTMS group, $P < 0.05$
